# Pectoral nerve block combined with general anesthesia for breast cancer surgery: a retrospective comparison

**DOI:** 10.1186/s40981-015-0018-1

**Published:** 2015-09-23

**Authors:** Harue Morioka, Yoshinori Kamiya, Takayuki Yoshida, Hiroshi Baba

**Affiliations:** 1 Division of Anesthesiology, Niigata University Graduate School of Medical and Dental Sciences, 1-757 Asahimachi-dori, Chuo Ward, Niigata, 951-8510 Japan; 2 Present address: Department of Anesthesiology, Niigata City General Hospital, 463-7 Shumoku, Chuo Ward, Niigata, 950-1197 Japan; 3 Present address: Department of Anesthesiology, Uonuma Institute of Community Medicine, Niigata University Medical and Dental Hospital, 4132 Urasa, Minami-Uonuma, Niigata 949-7302 Japan

## Abstract

**Background:**

Acute postoperative pain is an integral risk factor in the development of chronic pain after breast cancer surgery (BCS). Pectoral nerve block (PECSB) has been recently reported as an analgesic method for BCS. Here, we retrospectively compared intraoperative opioid requirement, postoperative pain after BCS, and incidence of postoperative nausea and vomiting (PONV) in patients who underwent BCS under total intravenous anesthesia (TIVA) with or without PECSB.

**Findings:**

We reviewed anesthesia charts and medical records of 146 patients who underwent BCS at Niigata University Medical and Dental Hospital from January 2013 to March 2014; 36 patients were included in the TIVA group, and 35 patients were included in the TIVA + PECSB group. Intraoperative remifentanil requirements were significantly lower in the TIVA + PECSB group than in the TIVA group, and the cumulative distribution of remifentanil was reduced in patients who received PECSB (TIVA: 10.9 ± 2.9 μg/kg/h; TIVA + PECSB: 7.3 ± 3.3 μg/kg/h; *p* < 0.001). Postoperative pain scores during the 48 h after surgery were significantly lower in the TIVA + PECSB group than in the TIVA group (TIVA: 2 [1–5]; TIVA + PECSB: 1 [0–5]; *p* = 0.03). However, administration of fentanyl during operation, percentage of patients requiring supplemental analgesics, and incidence of PONV were not significantly different between groups.

**Conclusions:**

PECSB significantly reduced intraoperative remifentanil usage and postoperative pain. However, the requirement for postoperative supplemental analgesics and the incidence of PONV did not differ. These data suggested that PECSB may be useful for perioperative pain management in patients undergoing BCS.

## Findings

### Introduction

Breast cancer is the most common cancer among women, and the incidence of breast cancer continues to rise. Acute postoperative pain is an integral risk factor in the development of chronic pain after breast cancer surgery (BCS); 40 % of women will have severe acute postoperative pain after BCS [1, 2], potentially disrupting the quality of postoperative recovery.

Thoracic paravertebral block (TPVB) is widely used for anesthesia and postoperative pain management for BCS [3–5]. However, TPVB is generally performed before general anesthesia for surgery, and not all anesthesiologists feel comfortable using such invasive techniques for BCS. Recently, Blanco et al. reported the use of pectoral nerve block (PECSB) as a new technique during BCS [6, 7]. PECSB is an interfascial plane block where local anesthetic is deposited into the plane between the pectoralis major muscle and the pectoralis minor muscle (PECS-I block) and above the serratus anterior muscle at the third rib (PECS-II block); blocking the pectoral; intercostobrachial; intercostals III, IV, V, and VI; and long thoracic nerves by PECSB is expected to provide analgesia for BCS [6, 7]. Moreover, PECSB is thought to be less invasive because it can be performed with the patient in the supine position and enables patients to undergo BCS after induction of general anesthesia. Interestingly, Bashandy and Abbas recently reported that PECSB was able to reduce intraoperative fentanyl requirement, postoperative pain, postoperative morphine consumption, and postoperative nausea and vomiting (PONV) in patients undergoing BCS [8].

In this study, we hypothesized that PECSB in patients undergoing BCS may be beneficial for reduced intra- and postoperative analgesics; we retrospectively analyzed anesthesia charts and medical records of patients who underwent BCS under total intravenous anesthesia (TIVA) with or without PECSB.

### Methods

#### Patients

This retrospective study was reviewed and approved by the institutional review board of Niigata University Medical and Dental Hospital (approved number: 1855 and trial registration number: UMIN000017875 [Efficacy of pectoral nerve block for perioperative pain management in breast cancer surgery], https://upload.umin.ac.jp/cgi-open-bin/ctr/ctr.cgi?function=brows&action=brows&recptno=R000020706&type=summary&language=E). We reviewed the anesthesia charts and medical records of all patients who underwent BCS under general anesthesia at Niigata University Medical and Dental Hospital from January 2013 to March 2014. During this period, we began to introduce PECSB for BCS, and PECSB was carried out if the anesthesiologists who were assigned to BCS were capable of performing this technique and the patient consented. Patients were excluded if they were male, underwent reconstructive surgery, underwent bilateral surgery, were a participant in an investigational new drug trial, received another nerve block (such as TPVB or epidural anesthesia), or underwent general anesthesia using inhaled anesthetics. Patients with incomplete medical records were also excluded from this study.

#### Analgesic methods and data collection

General anesthesia was induced and maintained with propofol and remifentanil. The dose of intraoperative propofol was controlled by target-controlled infusion to maintain a bispectral index (BIS) between 35 and 60. Intraoperative remifentanil was adjusted to maintain adequate anesthetic depth and systolic blood pressure (80–140 mmHg). Fentanyl was used for supplemental intraoperative analgesia if more than 0.5 μg/kg/min remifentanil was needed to maintain adequate anesthetic depth and systolic blood pressure or for transitional postoperative analgesia as needed. After induction of general anesthesia, PECS-I and PECS-II blocks were performed as previously described with 0.25 or 0.375 % ropivacaine or 0.25 or 0.5 % levobupivacaine under ultrasound visual guidance. We used 10–20 mL of local anesthetic for PECS-I block and 20–40 mL of local anesthetic for PESC-II block [6, 7]. Postoperative supplemental analgesics were administered if the patients felt more than mild postoperative pain (numerical rating scale (NRS) ≥4). For patients who did not start oral intake, a suppository of diclofenac sodium or intravenous flurbiprofen axetil was chosen, whereas for patients who started oral intake, loxoprofen sodium was chosen. Patient age, height, weight, body mass index (BMI), target propofol concentration for anesthesia maintenance, intraoperative opioid (fentanyl and remifentanil) administration, maximal postoperative pain score with NRS (0–10 at 07:00, 12:00, and 18:00 or at the time the patient requested supplemental analgesia during the first 48 h after surgery), postoperative supplemental analgesic (diclofenac sodium, flurbiprofen axetil, or loxoprofen sodium) administration, and PONV incidence during the first 48 h after surgery were extracted from the anesthesia charts and medical records of each patient.

#### Data analyses

Data for continuous variables were presented as the mean ± standard deviation (SD), and data for categorical or ordinal variables, as well as data that did not obey the normal distribution, were presented as the median and range. Data for continuous variables and data that obeyed the normal distribution were analyzed by paired *t* tests or one-way analysis of variance (ANOVA). Data for categorical variables or data that did not obey the normal distribution were analyzed by Mann-Whitney *U* tests and Fisher’s exact tests. The differences in distributions between patients undergoing BCS with or without PECSB were analyzed by Kolmogorov-Smirnov (K-S) tests. All statistical analyses were performed using Microsoft Excel 2011 for Macintosh (Microsoft, Redmond, WA) with a statistical macro (XLSTAT2014; Addinsoft, New York, NY). All *p* values were two-sided. Differences with *p* values of less than 0.05 were considered significant. The primary outcomes were intraoperative opioid requirement and postoperative pain during 48 h after BCS, and the secondary outcomes were postoperative supplemental analgesic use and PONV incidence during 48 h after BCS.

### Results

During the study period, 146 patients underwent BCS. We excluded patients with incomplete records (*n =* 27), those who underwent bilateral surgery or simultaneously had reconstructive surgery (*n =* 15), those who received another block (TPBV or epidural anesthesia; *n =* 15) or inhaled anesthesia (*n =* 17), and those participating in an investigational new drug trial (*n =* 1). In total, 36 patients were included in the TIVA group, and 35 patients were included in the TIVA + PECSB group.

**Fig. 1:**
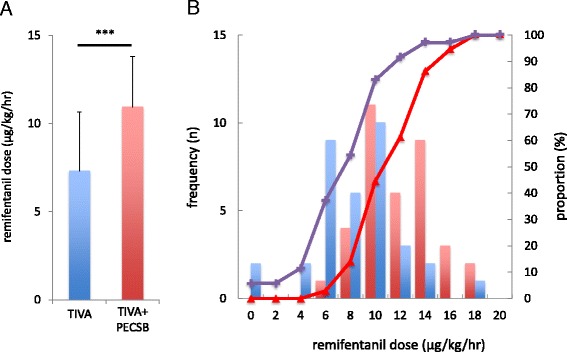
**a** Average dose of intraoperative remifentanil use in the TIVA and TIVA + PECSB groups. Data are shown as the means ± SDs. ***p < 0.001 by unpaired Student’s *t* tests. **b** Histogram and cumulative probabilities of intraoperative remifentanil dose in patients who underwent BCS with TIVA or TIVA + PECSB. *Red* and *blue bars* denote the frequency of increases in intraoperative remifentanil doses (2 μg/kg/h) in the TIVA and TIVA + PECSB groups, respectively. *Red triangles* and *purple crosses* denote the cumulative distribution of intraoperative remifentanil doses in the TIVA and TIVA + PECSB groups, respectively

Demographic data (age and BMI), duration of anesthesia and surgery, and target propofol concentration for maintenance were not different between the two groups (Table 1). Mean intraoperative remifentanil requirement was significantly lower in the TIVA + PECSB group than in the TIVA group, and cumulative distribution of remifentanil requirement was shifted to the right in patients receiving PECSB (TIVA: 10.9 ± 2.9 μg/kg/h; TIVA + PECSB: 7.3 ± 3.3 μg/kg/h; *p* < 0.001 by Student’s *t* test and K-S test; Fig. 1). However, intraoperative fentanyl administration was not different between groups (TIVA: 0.84 [0–6.85] μg/kg/h; TIVA + PECSB: 0 [0–7.57] μg/kg/h; *p* = 0.11).

**Table 1:** Demographic data, duration of anesthesia and surgery, and target propofol concentration for anesthesia maintenance

	TIVA (*n =* 36)	TIVA + PECSB (*n =* 35)	*p* value
Age (years)	60.6 ± 10.9	56.0 ± 12.7	0.11
Body mass index (kg/m^2^)	23.8 ± 3.8	22.5 ± 2.8	0.11
Duration of anesthesia (min)	159 ± 47	165 ± 34	0.53
Duration of surgery (min)	108 ± 43	106 ± 35	0.81
Target propofol concentration (μg/mL)	2.83 ± 0.46	2.95 ± 0.37	0.23

Data are shown as means ± SDs. Unpaired *t* tests were used to analyze the data

**Fig. 2:**
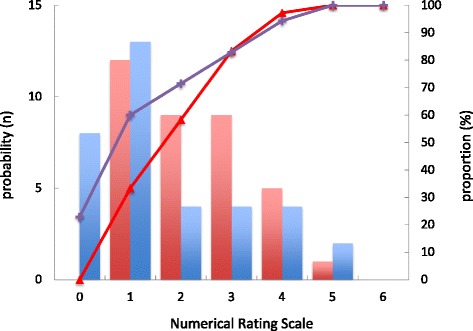
Histogram and cumulative probabilities of maximal postoperative pain during the 48 h after surgery in patients who underwent BCS with TIVA or TIVA + PECSB. *Red* and *blue bars* denote the frequencies of NRS in the TIVA and TIVA + PECSB groups, respectively. *Red triangles* and *purple crosses* denote cumulative distributions of NRS within the 48 h after surgery in the TIVA and TIVA + PECSB groups, respectively

Maximal postoperative NRS during the 48 h after surgery was significantly lower in the TIVA + PECSB group than in the TIVA group, and the cumulative distribution of NRS was shifted to the right in patients receiving PECSB (TIVA: 2 [1–5]; TIVA + PECSB: 1 [0–5]; *p* = 0.03 by Mann-Whitney *U* test and *p* = 0.04 by K-S test; Fig. 2). However, the percentage of patients who required supplemental analgesics (diclofenac sodium, flurbiprofen axetil, or loxoprofen sodium) was not different between the two groups (TIVA: 24.3 % [9/36]; TIVA + PECSB: 17.1 % [6/35]; *p* = 0.32). Additionally, the incidence of PONV was not different between the two groups (TIVA: 16.7 % [6/36]; TIVA + PECSB: 11.4 % [4/35]; *p* = 0.39).

### Discussion

In this retrospective study, we showed that intraoperative remifentanil administration and postoperative surgical pain during the 48 h after surgery in patients undergoing BCS could be reduced by PECSB. These results suggested that PECSB was useful for suppression of intraoperative nociception and reduction of postoperative pain during the early period after surgery.

Compared to TPVB, PECSB is thought to be safer and less invasive because PECSB is a type of superficial interfascial plane block and can be performed under ultrasound guidance [6–8]. Consistent with a recent report [8], total intraoperative opioid requirement and postoperative pain were reduced in the TIVA + PECSB group compared with those in the TIVA group.

However, in contrast with another recent report, intraoperative fentanyl consumption, postoperative supplemental analgesic requirement, and the incidence of PONV were unchanged, regardless of PECSB. These discrepancies may be explained by the following observations. We used fentanyl as an analgesic for transitional analgesia in anticipation of pain relief during the early postoperative period; therefore, similarities in fentanyl consumption may have affected the results. Additionally, there was insufficient statistical power for analyzing differences in fentanyl consumption.

There was no inter-group difference in the incidence of PONV in this study, even though the dose of intraoperative remifentanil in the TIVA + PECSB group was significantly lower than that in the TIVA group. Some studies indicated that doses of both fentanyl and remifentanil during surgery were risk factors for PONV [9, 10], but other studies showed that intraoperative remifentanil dose did not correlate with the severity of PONV [11, 12]. This study suggests that intraoperative dose of remifentanil may not affect the incidence of PONV. However, the etiology of PONV is multifactorial, and there are several risk factors other than perioperative opioid use, homogenous gender group, shorter duration of anesthesia, and avoidance of volatile anesthetics. Therefore, the inhomogeneous background of the risk factors of PONV in this study may influence the insignificant incidence of PONV. Further extensive study is needed to draw a solid conclusion about the correlation between the lesser opioid consumption with PECS block and the incidence and severity of PONV after breast surgery.

The lack of significant differences in the administration of postoperative supplemental analgesics may be explained by the following observations. PECSB cannot block the anterior cutaneous branches of the intercostal nerves, which innervate the nearby sternum; therefore, the internal mammary region in the surgical site may not be blocked by PECSB [13], although infusion of remifentanil plus fentanyl would still be expected to suppress intraoperative nociception. Moreover, pain was treated on a case-by-case basis rather than using a standard protocol.

Our study had several limitations. First, a variety of intraoperative analgesics were used for each patient. Second, the concentration and volume of local anesthetics for PECSB were not standardized among cases. Finally, intraoperative antiemetic drug usage for prevention of PONV was also not rigorously standardized; this may have affected the observed incidence of PONV. Further prospective, case-control studies are required in order to overcome these limitations.

### Conclusion

In this study, we evaluated the efficacy of PECSB for BCS. PECSB significantly reduced intraoperative remifentanil usage and postoperative pain. However, the requirement for postoperative supplemental analgesics and the incidence of PONV did not differ between the two groups. Although a more in-depth analysis with a prospective study design is needed, our analysis supported that PECSB reduced postoperative pain to some extent in patients undergoing BCS.

## Abbreviations

BCS: breast cancer surgery
BMI: body mass index
K-S test: Kolmogorov-Smirnov test
PECSB: pectoral nerve block
PONV: postoperative nausea and vomiting
TIVA: total intravenous anesthesia
